# AGE-BASED STEREOTYPE THREAT: A SYSTEMATIC REVIEW OF PRIMING TECHNIQUES AND THEIR EFFECTS

**DOI:** 10.1093/geroni/igz038.1705

**Published:** 2019-11-08

**Authors:** Francesco Vailati Riboni, Francesco Pagnini

**Affiliations:** Università Cattolica del Sacro Cuore di Milano, Milan, Italy, Italy

## Abstract

Age-based stereotype threat (ABST) occurs when older adults are influenced by negative stereotypes about age-related decline and functional losses and ironically behave in disengaging and self-defeating ways that confirm the stereotype (Steele & Aronson, 1995). Aging stereotypes are found to be strong predictors of health and illness outcomes in later life, and are associated with performance in specific areas, mainly in cognitive and physical domains. The current study reviewed the experimental methods and their reported effects previously published in the literature to determine if there were different ABST methods were associated with different types of age-related outcomes. We conducted a systematic review, screening the scientific literature for papers that included experimental manipulation of age-related stereotypes as an independent variable, focusing on samples of older adults (1113 articles, most published after 2003). Through a classification of the common and distinctive characteristics of the different stereotype manipulation techniques, we were able to identify three specific types of experimental methods: by instruction, tests, and interpersonal exposure. Although the mechanism by which stereotypes are associated with functional outcomes in older adults remains unclear, our review suggests it is possible to experimentally control the activation of the stereotype by manipulating its specific characteristics and the way older participants are exposed to it. Findings also highlight the possibility that specific experimental methods used to induce ABST in older individuals may lead to unique and different consequences on functional performance variation.

